# Understanding the Uptake and Outcomes of Non-occupational Postexposure Prophylaxis Use Through an Online Medical Platform in China: Web-Based Cross-sectional Study

**DOI:** 10.2196/42729

**Published:** 2023-05-19

**Authors:** Duo Shan, Hui Xue, Fei Yu, Xingkai Zan, Hui Liu, Jiaye Liu, Mengjie Han, Dapeng Zhang

**Affiliations:** 1 National Center for AIDS/STD Control and Prevention Chinese Center for Disease Control and Prevention Beijing China; 2 Danlan Goodness Beijing China; 3 School of Public Health Shenzhen University Health Science Center Shenzhen China

**Keywords:** online medical platform, men who have sex with men, postexposure prophylaxis, preexposure prophylaxis, PrEP, sexual behaviors, HIV, risk, prevention

## Abstract

**Background:**

To date, non-occupational postexposure prophylaxis (PEP) has been widely accepted as a safe and effective intervention for HIV in many countries, yet it remains an underutilized prevention strategy in China. Evidence indicated a high demand for PEP among Chinese men who have sex with men, but the uptake and access to PEP service remain limited. In an era of rapid development of web-based technology, online medical platforms in China hold great promise in facilitating PEP provision and delivery by addressing problems such as accessibility, convenience, privacy protection, and antidiscrimination by integrating online and offline resources. However, there is a paucity of data concerning the uptake and outcomes of online PEP in China.

**Objective:**

The aim of this study is to explore online PEP service provision and understand PEP uptake and outcome through a web-based cross-sectional study.

**Methods:**

From January 2020 to June 2021, we conducted a retrospective web-based survey among those seeking online PEP services via the internet medical platform “HeHealth” using a structured questionnaire. Participants were surveyed on sociodemographic characteristics, sexual and drug-related behaviors, history of preexposure prophylaxis (PrEP) usage, and PEP uptake. Statistical analysis included descriptive analysis, chi-square test, and multivariable logistic regression. *P* values <.05 were deemed statistically significant.

**Results:**

No HIV seroconversions were observed among 539 PEP users. Our sample demonstrated that most participants seeking online PEP services were gay (397/539, 73.7%), single (470/539, 87.2%), having an education of more than 12 years (493/539, 91.5%), and with an average monthly income of 7000 RMB (1 RMB=US $0.14) or more (274/539, 50.8%). Sexual exposures accounted for 86.8% (468/539) of the cases, with anal sex being the most common indication (389/539, 72.2%) for seeking PEP use. Among 539 participants, 60.7% (327/539) sought online PEP for relatively low-risk exposures, whereas 39.3% (212/539) were considered high-risk exposures. Nearly all (537/539, 99.6%) initiated PEP within 72 hours and 68.6% (370/539) within 24 hours of exposure. All users (539/539) were prescribed a 3-drug regimen, with most comprising 3TC/TDF+DTG (lamivudine, tenofovir disoproxil fumarate, and dolutegravir; 293/539, 54.4%), followed by FTC/TDF+DTG (emtricitabine, tenofovir disoproxil fumarate, and dolutegravir; 158/539, 29.3%). The adjusted model showed that greater odds of PrEP usage were associated with an age of 35 years or older versus the age group of 25-34 years (adjusted odds ratio [AOR] 2.04, 95% CI 1.24-3.37), having an education of 17 years or more versus an education of 12 years or less (AOR 3.14, 95% CI 1.29-7.62), average monthly income of 20,000 RMB or more versus less than 3000 RMB (AOR 2.60, 95% CI 1.09-6.23), and having high-risk sexual behavior during PEP treatment (AOR 2.20, 95% CI 1.05, 3.69).

**Conclusions:**

The 0% infection rate in this study demonstrated that online PEP could be a valuable risk-reduction option to improve HIV prevention service within China. However, further research is needed to better facilitate PrEP transition among online PEP users.

## Introduction

### Background

HIV continues to be a major public health concern worldwide. In 2020, approximately 37.7 million people were living with HIV globally, with 1.5 million new infections reported by the end of 2020 [1]. The Joint United Nations Program on HIV/AIDS (UNAIDS) showed that key populations and their sexual partners accounted for 65% of the HIV infections [2]. In China, based on the national HIV/AIDS case reporting system, there were 1.05 million people living with HIV by the end of 2020 and over 60,000 new cases were reported in the first half of 2021 [3]. In nearly every country with available data, HIV prevalence among men who have sex with men (MSM) exceeds that within the general population [4,5].

In China, the proportion of HIV infection through male-to-male sexual contacts increased from 9.1% in 2009 to 23.3% in 2020 [6-8]. In an effort to reduce HIV infection and transmission among MSM, China has responded actively by scaling up condom use, HIV counseling and testing, and other behavioral interventions. However, data based on national HIV sentinel surveillance showed that HIV prevalence among MSM fluctuated around 8% in the last 5 years, which has been far higher than that of other high-risk groups [3,9]. In fact, the HIV epidemic among MSM has become the top challenge of HIV response in China.

Given the persistent high HIV prevalence among MSM, it seems that current strategies are far from sufficient to reduce HIV transmission among this population. As a biomedical HIV prevention strategy, nonoccupational HIV postexposure prophylaxis (PEP) has been proven to be a safe and effective strategy aimed at preventing HIV among those with a recent HIV exposure [10-13]. Currently, PEP has been widely implemented in many countries [14-17]; however, it remains an underutilized prevention strategy in China [18,19]. Between 2018 and 2019, the National Center for AIDS/STD Control and Prevention, Chinese Center for Disease Control and Prevention (NCAIDS, China CDC) carried out a pilot program of PEP among MSM in 7 provinces to promote the uptake of PEP and preexposure prophylaxis (PrEP) [20]. Later, China released the Implementation Plan to Curb the Spread of AIDS (2019-2020), which encouraged the application of PEP programs. Yet, due to the lack of sufficient research data, the Chinese Guideline on HIV PEP has not been formulated until October 2020 [21]. To date, the few published studies on HIV PEP in China have mainly focused on awareness and willingness to take PEP among high-risk populations, which showed that over one-half of the MSM were aware of PEP, with more than 60% highlighting the need for improved PEP access [19,22,23]. The findings of several other pilot studies in China also showed a high demand for PEP among MSM [24-28]. However, as PEP services are mainly provided in designated hospitals for AIDS treatment in China, it has not yet been promoted and applied on a large scale. To obtain PEP drugs, people need to go to the designated hospitals and get a prescription from doctors first, which often lack flexibility in visiting hours and location selection; additionally, there were concerns among clients regarding the lack of confidentiality and access to trained health care providers on consultation and care around PEP [29,30]. Actually, PEP was inaccessible to many due to various reasons, such as passive waiting for services in hospitals, no specialized clinic for PEP service, and part-time medical staff providing PEP services, among others, all of which led to the contradiction between the urgent demand for PEP services among MSM and the lack of corresponding medical support in China.

Under these circumstances, it may be best to leverage other available resources and explore new modalities to help facilitate PEP use. In short, exposed populations who have a potential risk of HIV infection would be more likely to benefit from PEP if they know where to receive convenient PEP services and are provided with appropriate drugs. Owing to the development of internet technology and the rise in popularity of web-based social and sexual networking in recent years, Chinese social media platforms have been not only helping expand social circles and build community, but also providing mediums for acquiring new information, thereby making online HIV intervention possible [31]. Recently, it has also been recommended to make full use of social media and internet medical platforms to strengthen health education on PEP and further promote HIV PEP uptake. In December 2021, in order to improve the accessibility and standardization of “Internet plus HIV prevention services,” the “Expert consensus on developing HIV-related medical services in internet hospitals” was developed to further standardize internet-based diagnosis and treatment, accelerating the process of internet plus HIV PEP service [32].

A review of available literature shows that there is paucity of data concerning the suitable provision and delivery models of HIV PEP services in China despite the high prevalence of HIV among MSM [33]. Moreover, PEP could serve as an opportunity to identify individuals eligible for PrEP [34,35], which has been an equally promising and effective tool to prevent HIV transmission [36,37]; however, there is a lack of data on factors of PrEP usage among PEP users in China.

### Study Objectives

To address these gaps and make PEP service more accessible, the NCAIDS, China CDC collaborated with “HeHealth,” an online health service platform embedded in Blued, the most popular geosocial networking app for gay people in China [38], to explore the modality of online PEP services provision among Chinese MSM. The goal of this study was to gain insight into the following: (1) characteristics of online PEP recipients, (2) uptake and outcomes of online PEP use, and (3) factors of PrEP usage among those seeking PEP services through the internet.

## Methods

### Study Design and Participants

We conducted a retrospective cross-sectional study inviting those who ever used PEP services via the “HeHealth” internet medical platform to participate in the survey. A survey invitation was sent to all users of “HeHealth” who received PEP services between January 2020 and June 2021. After providing informed consent, participants completed an anonymous online questionnaire. Before submission, participants could review all items of the questionnaire and make sure mandatory items were completed (see Multimedia Appendix 1 [39]). Participants who completed the questionnaires within 30 seconds or submitted incomplete questionnaires were considered likely to be untruthful or not serious and were thus excluded. Each internet protocol address was restricted to filling out only 1 questionnaire. Participants were eligible for the study if they were aged over 18 years and ever used online PEP service via “HeHealth.” All participant data were deidentified prior to data analysis.

### PEP Services Provided by “HeHealth”

HeHealth is an online medical platform [40] established by Blued (BlueCity Group) in 2019. Launched in 2012, Blued has become the most popular gay geosocial networking app in China, with more than 70 million registered users and over 7 million monthly active users, half of which are in mainland China. HeHealth, embedded in the Blued app, is committed to providing friendly, convenient, and confidential HIV prevention and intervention solutions. Besides Blued users, other people at risk for HIV can visit HeHealth through channels such as WeChat (Tencent Holdings Limited), Microblog (Sina Corporation), Zhihu (Zhihu Inc.), Douyin (ByteDance Ltd.), and Bilibili (Bilibili, Inc.). Users can access the platform for HIV knowledge, HIV/syphilis/hepatitis B virus (HBV) testing support, online counseling, online diagnosis and prescription by contracted doctors, drug delivery, PrEP and PEP, and follow-up services. Until June 2022, nearly 50 doctors and specialists in AIDS/sexually transmitted disease have registered in HeHealth. For PEP users, the services provided by the platform are listed in Textbox 1.

**Services provided by the online medical platform HeHealth.**

**1. Online counseling**

Users can log into the platform and approach a doctor for postexposure prophylaxis (PEP) counseling after completing a questionnaire collecting related behavioral information that helps doctors to evaluate their risk.

**2. Online medication**

After evaluation based on communication between the user and the doctor, if eligible for PEP, the user will receive a prescription of a 28-day antiretroviral regimen consisting mainly of 2 nucleos(t)ide reverse transcriptase inhibitors and a third agent (protease inhibitor or integrase inhibitor) as well as necessary medical checks.

**3. Service arrangement**

A health management assistant (usually a practicing pharmacist) will then call the user to consult on the use of PEP drugs, related tests, and most importantly the fast way to deliver the drugs.

**4. Package delivery**

Once confirmed, the prescription will be transferred to the most convenient drug store (>100 contracted drug stores in 80 major cities across China) and a PEP service package will be delivered (mostly via flash delivery) by the specific drug store. The package contains finger prick test kits for HIV, hepatitis B virus, and syphilis; PEP drugs; and a PEP instruction manual.

**5. Follow-up services**

**Figure 1:**
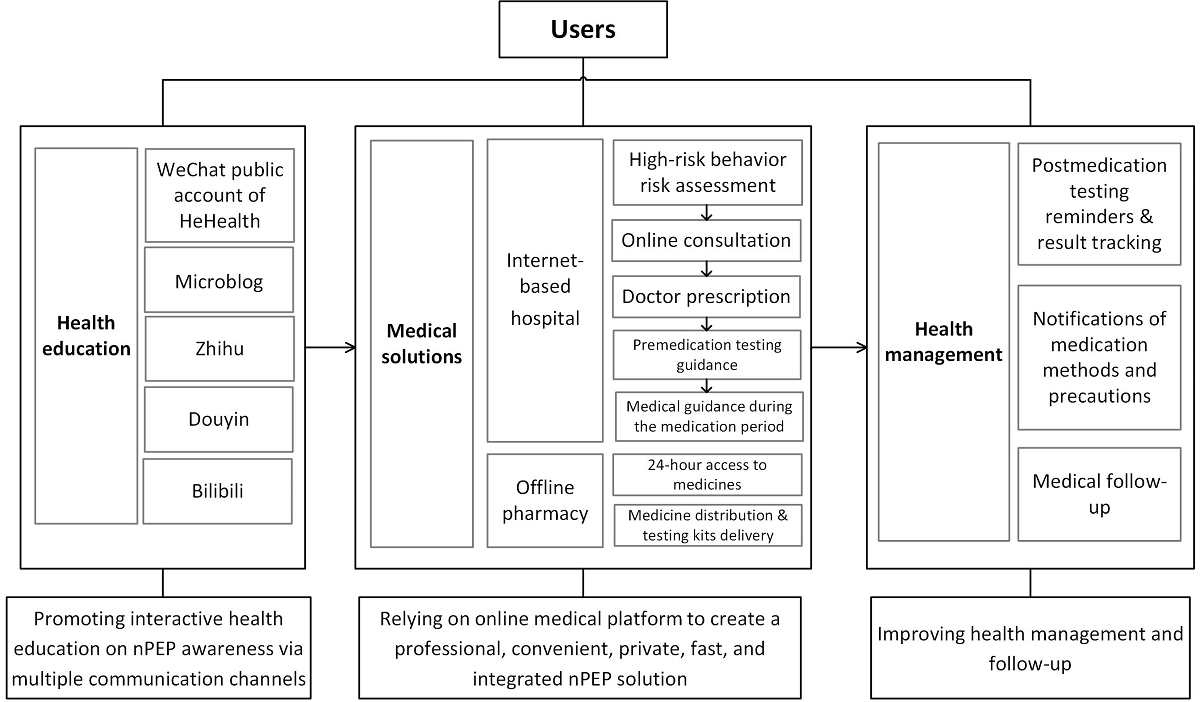
Online and offline PEP service processes of HeHealth (Blued). nPEP: nonoccupational HIV postexposure prophylaxis.

For those who initiated PEP, the platform will provide a free 28-day 1-on-1 doctor consultation and follow-up visits via a phone call at week 2, with HIV test reminders at weeks 4, 12, and 24. Figure 1 shows the whole online and offline PEP service process of HeHealth.

### Ethics Approval

The study protocol was reviewed and approved by the Institutional Review Board Committee of National Center for AIDS/STD Control and Prevention, Chinese Center for Disease Control and Prevention (X220511685).

### Measures

#### Participant Sociodemographic Characteristics

We collected the following participant characteristics: gender, sexual orientation, age, marital status, educational status, work status, and average monthly income (in Chinese Yuan).

#### Sexual and Drug-Related Behaviors

Participants were asked about their sexual behavior, including engagement in sex (anal, fellatio, receptive, or insertive) and their role (ie, insertive or receptive; participants were able to select both roles if applicable) at the time of exposure; condom use; type of the source contact (eg, casual partner, regular partner, commercial partner, and others); and HIV status of the source contact prior to the exposure (positive, negative, or unknown). Participants were also asked about recent (past 6 months) engagement in recreational drug use before or during sexual activity. Recreational drug use includes amyl nitrate (poppers), crystal methamphetamine, cocaine, heroin, or ecstasy. Additionally, participants were asked if they had ever been tested for or diagnosed with HIV or other sexually transmitted infections before initiating PEP, including syphilis, gonorrhea, hepatitis B, and hepatitis C.

#### Exposure Risk Assessment

High- and low-risk exposures were discerned specifically from a public health perspective. According to the Chinese Guideline on HIV PEP, relatively high-risk exposure conditions included the following: (1) risk event including receptive or insertive anal sex; (2) did not use a condom, experienced condom breakage/slippage/removal, or others; and (3) source contact was highly suspected to be HIV positive for multiple reasons, was known to be HIV positive, or was of unknown HIV status; among those with source contact that was HIV positive, the scenarios included not under treatment, treatment status unknown, and under treatment with viral nonsuppression. Relatively low-risk exposure conditions included the following: (1) risk event did not include receptive or insertive anal sex; (2) used a condom; (3) source contact was known to be HIV negative, or was HIV positive but undertreatment and virally suppressed.

#### PEP and PrEP Usage

We collected information on PEP regimen prescribed, problems during medication, HIV high-risk sexual behavior during PEP treatment, and prior use of PEP. PEP completion was defined as self-reported completion of a 28-day course therapy. We hypothesized that a certain proportion of participants presenting for PEP would be candidates for PrEP based on the current Chinese Guideline on HIV PEP, with participants considered as PrEP candidates if they ever used PrEP.

### Data Analysis

Analyses were conducted using SPSS version 26.0 (IBM, Inc.). Frequency distribution and percentages were calculated for categorical variables, whereas median and interquartile range (IQR) were calculated for continuous variables with nonnormal distribution. For variables with a missing ratio of less than 5%, we imputed missing values in the database by mean for the variables “the time from exposure to decision of taking PEP,” “time from the decision to submission of online medication requests,” and “time for drugs delivery,” and conducted the same analysis using list-wise deletion to determine whether imputation affected the study findings. We used univariable logistic regression to calculate odds ratios (ORs) and their 95% CIs for potential factors associated with PrEP candidacy among PEP users. We further analyzed factors of PrEP candidacy using multivariable logistic regression analysis through stepwise elimination to adjust for confounders, and adjusted ORs (AORs) with 95% CIs were calculated. Variables that were found to be either significantly or marginally associated with PrEP usage in the univariate analysis were considered for inclusion in the multivariate model, together with those from a priori hypothesis from the related literature. These included region, age, education, average monthly income, HIV high-risk sexual behavior during PEP treatment, and prior use of PEP [37]. Variables with 2-tailed *P*<.05 were considered statistically significant.

## Results

### Sociodemographic and Behavioral Characteristics

Between January 2020 and June 2021, a total of 5001 users with a history of accessing PEP drugs via “HeHealth” clicked on the survey invitation link and reached the landing page of the survey. Of these, 716 (14.32%) provided informed consent and completed the survey. After excluding questionnaires with inconsistent information or core logic errors, 539 participants were finally included as the study sample. The demographics of the analytical sample (N=539) are presented in Table 1. Except Tibet, participants came from all 30 provinces in China. The top 5 provinces in the number of respondents were Guangdong (83/539, 15.4%), Beijing (56/539, 10.4%), Jiangsu (34/539, 6.3%), Shanghai (34/539, 6.3%), and Sichuan (33/539, 6.1%). A majority of the participants were gay (397/539, 73.7%), single (470/539, 87.2%), and with an education of 12 years or more (493/539, 91.5%). Their median age was 27 years (range 19-58 years). Over one-half (274/539, 50.8%) of the participants had an average monthly income of 7000 RMB (equivalent to US $980; 1 RMB=US $0.14) or more.

Sexual exposures accounted for 86.8% (468/539) of all PEP users, with anal sex being the most common indication (389/539, 72.2%) for seeking PEP use. Only 36.5% (142/389) used condom throughout anal sex and 79.4% (309/389) of the partners were casual partners. Drug use at the time of exposure was recorded for 38.2% (206/539) of the participants, with inhalant nitrites or “rush poppers” being most commonly used (194/206, 94.2%; Table 1).

**Table 1:** Sociodemographic and behavioral characteristics of online PEP^a^ users (N=539).

Variables				Values, n (%)
**Demographics**				
	**Gender**			
		Male	539 (100.0)	
	**Sexual orientation**			
		Homosexual	397 (73.7)	
		Bisexual	120 (22.3)	
		Heterosexual and unknown	22 (4.1)	
	**Marital status**			
		Divorced/widowed	15 (2.8)	
		Single	470 (87.2)	
		Married/cohabiting	54 (10.0)	
	**Age (years)**			
		19-24	114 (21.2)	
		25-34	327 (60.7)	
		35-44	83 (15.4)	
		45-58	15 (2.8)	
	**Education (years)**			
		≤12	46 (8.5)	
		13-16	382 (70.9)	
		≥17	111 (20.6)	
	**Work status**			
		Full-time worker	336 (62.3)	
		Student	98 (18.2)	
		Freelancer	78 (14.5)	
		Others	27 (5.0)	
	**Average monthly income (RMB^b^)**			
		<3000	84 (15.6)	
		3000-6999	181 (33.6)	
		7000-9999	106 (19.7)	
		10,000-19,999	120 (22.3)	
		≥20,000	48 (8.9)	
	**China location**			
		Eastern	333 (61.8)	
		Western	98 (18.2)	
		Central	108 (20.0)	
**HIV-related risk behaviors**				
	**Type of exposure**			
		Anal sex	389 (72.2)	
		Only oral sex	79 (14.7)	
		Being bitten, scratched, and others	33 (6.1)	
		Only rubbing of genitals with no insertion	21 (3.9)	
		Others	17 (3.2)	
	**Condom use during anal sex at the time of exposure (n=389)**			
		Yes	142 (36.5)	
		No	174 (44.7)	
		Condom breakage/slippage/removal, or others	53 (13.6)	
		Missing	20 (5.1)	
	**Anal sex roles during exposure (n=389)**			
		Receptive	216 (55.5)	
		Insertive	130 (33.4)	
		Both	24 (6.2)	
		Refused to answer	19 (4.9)	
	**Type of source contact during exposure (n=389)**			
		Casual partner	309 (79.4)	
		Regular partner	58 (14.9)	
		Commercial partner	3 (0.8)	
		Others	19 (4.9)	
	**Recreational drug use in the past 6 months**			
		Rush poppers	194 (36.0)	
		Mixed use of drugs	12 (2.2)	
		No	333 (61.8)	
	**Diagnosed or treated for a** **sexually transmitted infection** **in the past 6 months**			
		Yes	22 (4.1)	
		No	517 (95.9)	
**Testing results before PEP use**				
	**HIV testing (** **n=485)**			
		Positive	0 (0.0)	
		Negative	485 (100.0)	
	**Hepatitis B testing** **(n=282)**			
		Positive	6 (2.1)	
		Negative	276 (97.9)	
	**Hepatitis C testing (** **n=121** **)**			
		Positive	0 (0.0)	
		Negative	121 (100.0)	
	**Syphilis testing (n=** **273)**			
		Positive	15 (5.5)	
		Negative	258 (94.5)	

^a^PEP: postexposure prophylaxis.

^b^1 RMB=US $0.14.

### Exposure Risk Assessment of Those Seeking Online PEP Services

**Figure 2:**
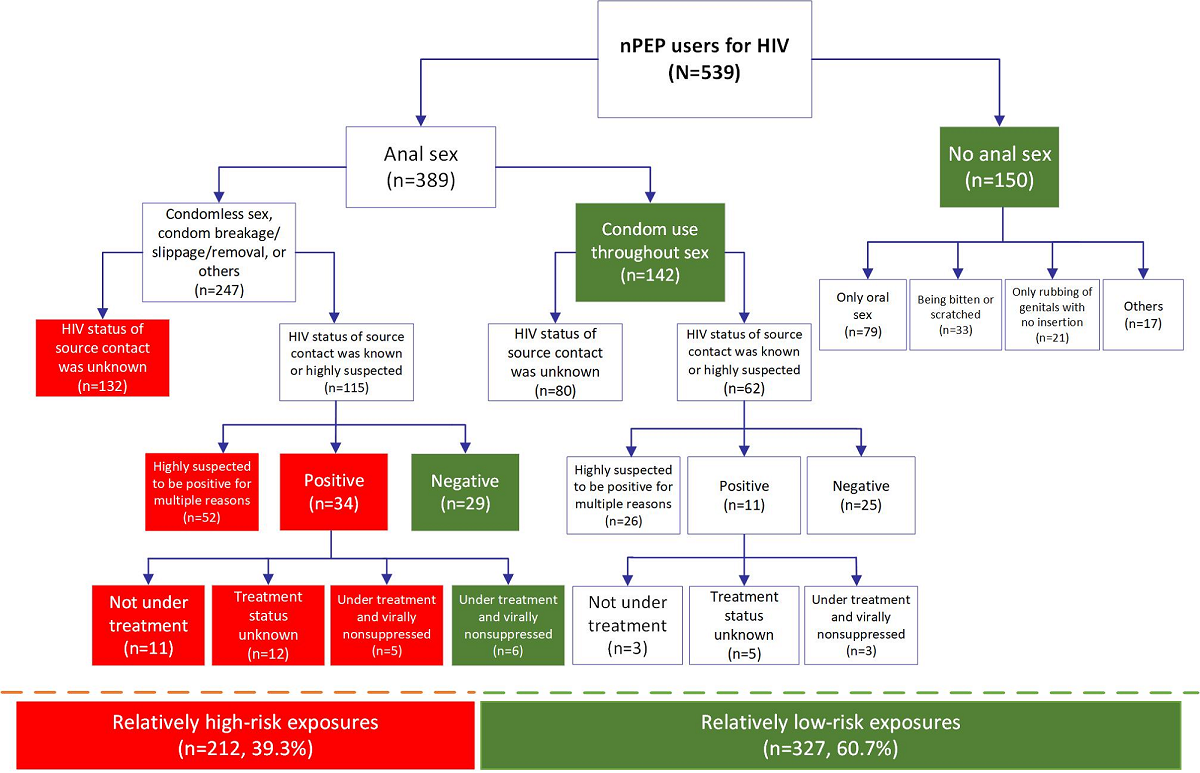
Exposure risk assessment of those seeking online PEP services according to the Chinese Guideline on HIV PEP. nPEP: nonoccupational HIV postexposure prophylaxis; PEP: postexposure prophylaxis.

A total of 327/539 (60.7%) participants sought online PEP for relatively low-risk exposures, including only oral sex (n=79), being bitten or scratched (n=33), only being rubbed of genitals with no insertion (n=21), condom use throughout anal sex (n=142), and exposures to HIV-negative sources (n=29) and HIV-positive sources under treatment with viral suppression (n=6); by contrast, 212/539 (39.3%) were considered to have experienced relatively high-risk exposures, including unprotected anal sex with source contacts whose HIV status was unknown (n=132), highly suspected to be positive (n=52), positive with no treatment (n=11), treatment status was unknown (n=12), and had treatment without viral suppression (n=5; Figure 2).

### HIV, Hepatitis B Virus, Hepatitis C Virus, and Syphilis Testing Prior to PEP Use

Most participants (485/539, 90.0%) received HIV testing prior to PEP use and none were seropositive. More than one-half (282/539, 52.3%) had HBV test, and the positive rate of HBV was 2.1% (6/282). Less than one-quarter (121/539, 22.4%) received hepatitis C virus (HCV) testing, and none tested positive before PEP use. A small portion of participants (15/273, 5.5%) tested positive for syphilis before PEP initiation.

### Uptake of PEP Services

Among 539 participants who sought the online PEP service, the time from exposure to PEP use ranged from 1 to 80 hours with a median time of 24 hours (IQR 10-36 hours). The majority of individuals (537/539, 99.6%) initiated PEP within 72 hours and 370 (68.6%) were within 24 hours of exposure. Specifically, the median time was 5 hours (range 1-76 hours) from exposure to decision of taking PEP, 1 hour (0-48 hours) from the decision to submission of online medication requests, and 6 hours (0-78 hours) for drugs delivery.

No HIV seroconversions were observed among the 539 PEP users. All users (539/539) were prescribed a 3-drug regimen consisting of 3TC/TDF+DTG (ie, lamivudine, tenofovir disoproxil fumarate, and dolutegravir) in most cases (293/539, 54.4%), followed by FTC/TDF+DTG (ie, emtricitabine, tenofovir disoproxil fumarate, and dolutegravir; 158/539, 29.3%). Almost all PEP users (523/539, 97.0%) self-reported to have completed the full 28-day course of medication and the remaining (16/539, 3.0%) stopped early. In addition, over one-quarter (140/539, 26.0%) utilized PEP prior to this exposure and 9.1% (49/539) had HIV high-risk sexual behaviors during PEP treatment, among which 51% (25/49) had been classified as high-risk exposure. A majority of individuals (396/539, 73.5%) were adherent to the treatment, and reasons for noncompliance included failure to take medicine at scheduled time (108/539, 20.0%) and forgetting to take medicines or taking them wrongly (35/539, 6.5%). No serious adverse drug reactions following the intake of PEP drugs were observed for the surveyed participants. Over one-third (177/539, 32.8%) ever initiated PrEP and all of them were willing to use PrEP. There were no statistical differences on PEP uptake among different risk-level groups (Table 2).

**Table 2:** PEP^a^ use by exposure risk level (N=539).

Variables			Total (N=539), n (%)		Risk levels					*P* value
					High (n=212), n (%)		Low (n=327), n (%)			
**PEP regimen prescribed**									.68	
	3TC^b^/TDF^c^+DTG^d^	293 (54.4)		121 (57.1)		172 (52.6)				
	FTC^e^/TDF+DTG	158 (29.3)		60 (28.3)		98 (30.0)				
	FTC/TAF^f^+DTG	42 (7.8)		14 (6.6)		28 (8.6)				
	FTC/TDF+RAL^g^	34 (6.3)		14 (6.6)		20 (6.1)				
	Others	12 (2.2)		3 (1.4)		9 (2.8)				
**PEP completion**									.38	
	Yes	523 (97.0)		204 (96.2)		319 (97.6)				
	No	16 (3.0)		8 (3.8)		8 (2.4)				
**Prior use of PEP**									.70	
	Yes	140 (26.0)		57 (26.9)		83 (25.4)				
	No	399 (74.0)		155 (73.1)		244 (74.6)				
**Problems during medication**									.39	
	No	396 (73.5)		159 (75.0)		237 (72.5)				
	Failure to take medicine on time	108 (20.0)		37 (17.5)		71 (21.7)				
	Taking wrongly or forgot to take medicine	35 (6.5)		16 (7.5)		19 (5.8)				
**HIV high-risk sexual behavior during PEP treatment**									.08	
	Yes	49 (9.1)		25 (11.8)		24 (7.3)				
	No	490 (90.9)		187 (88.2)		303 (92.7)				
**Ever initiated PrEP^h^**									.50	
	Yes	177 (32.8)		66 (31.1)		111 (33.9)				
	No	362 (67.2)		146 (68.9)		216 (66.1)				

^a^PEP: postexposure prophylaxis.

^b^3TC: lamivudine.

^c^TDF: tenofovir disoproxil fumarate.

^d^DTG: dolutegravir.

^e^FTC: emtricitabine.

^f^TAF: tenofovir alafenamide fumarate.

^g^RAL: raltegravir.

^h^PrEP: preexposure prophylaxis.

### Correlates of PrEP Usage Among Online PEP Users

Table 3 shows the results of binary logistic regression carried out to determine correlates associated with HIV PrEP usage among PEP users. In the univariate analysis, age, education, average monthly income, HIV high-risk sexual behavior during PEP treatment, and times of PEP use were significantly associated with HIV PrEP usage among PEP users (*P*<.05 for all variables; see Table 3 for precise *P* values).

The multivariable analysis indicated that, compared with those aged 25-34 years with an education of 12 years or less, an average monthly income of <3000 RMB, and not having HIV high-risk sexual behavior during PEP treatment, people aged 35 years or older (AOR 2.04, 95% CI 1.24-3.37) with an education of 17 years or more (AOR 3.14, 95% CI 1.29-7.62), having an average monthly income between 7000 and 9999 RMB (AOR 2.20, 95% CI 1.05-4.62) or 10,000-19,999 RMB (AOR 2.55, 95% CI 1.23-5.30) or 20,000 RMB or more (AOR 2.60, 95% CI 1.09-6.23), and having HIV high-risk sexual behavior during PEP treatment (AOR 2.20, 95% CI 1.05-3.69) had greater odds of PrEP usage among PEP users.

**Table 3:** Correlates of PrEP^a^ usage among 539 online PEP^b^ users.

Variables			Total (N=539), n (%)		PrEP usage					Crude odds ratio (95% CI)			*P* value			Adjusted odds ratio^c^ (95% CI)			*P* value
					No (n=362), n (%)		Yes (n=177), n (%)												
**Sexual Orientation**																			
	Heterosexual and unknown	22 (4.1)		14 (3.9)		8 (4.5)		1			—^d^			—			—		
	Bisexual	120 (22.3)		81 (22.4)		39 (22.0)		0.84 (0.33-2.18)			.72			—			—		
	Homosexual	397 (73.7)		267 (73.8)		130 (73.4)		0.85 (0.35-2.08)			.73			—			—		
**China location**																			
	Central	108 (20.0)		79 (21.8)		29 (16.4)		1			.14			—			—		
	Eastern	333 (61.8)		224 (61.9)		109 (61.6)		1.33 (0.82-2.15)			.25			—			—		
	Western	98 (18.2)		59 (16.3)		39 (22)		1.8 (1-3.24)			.05			—			—		
**Marital status**																			
	Married/cohabiting	54 (10.0)		32 (8.8)		22 (12.4)		1			—			—			—		
	Divorced/widowed	15 (2.8)		8 (2.2)		7 (4.0)		1.27 (0.40-4.02)			.68			—			—		
	Single	470 (87.2)		322 (89.0)		148 (83.6)		0.67 (0.38-1.19)			.17			—			—		
**Age (years)**																			
	25-34	327 (60.7)		224 (61.9)		103 (58.2)		1			—			1			—		
	13-24	114 (21.2)		88 (24.3)		26 (14.7)		0.64 (0.39-1.06)			.08			1.07 (0.61-1.89)			.81		
	≥35	98 (18.2)		50 (13.8)		48 (27.1)		2.09 (1.32-3.31)			<.001			2.04 (1.24-3.37)			.01		
**Education (years)**																			
	≤12	46 (8.5)		37 (10.2)		9 (5.1)		1			—			1			—		
	13-16	382 (70.9)		263 (72.7)		119 (67.2)		1.86 (0.87-3.98)			.11			2.14 (0.96-4.78)			.06		
	≥17	111 (20.6)		62 (17.1)		49 (27.7)		3.25 (1.43-7.37)			.01			3.14 (1.29-7.62)			.01		
**Work status**																			
	Freelancer and others	105 (19.5)		71 (19.6)		34 (19.2)		1			—			—			—		
	Full-time worker	336 (62.3)		214 (59.1)		122 (68.9)		1.19 (0.75-1.9)			.46			—			—		
	Student	98 (18.2)		77 (21.3)		21 (11.9)		0.57 (0.3-1.07)			.08			—			—		
**Average monthly income (RMB^e^)**																			
	<3000	84 (15.6)		68 (18.8)		16 (9.0)		1			—			1			—		
	3000-6999	181 (33.6)		135 (37.3)		46 (26.0)		1.45 (0.76-2.74)			.26			1.42 (0.72-2.82)			.31		
	7000-9999	106 (19.7)		67 (18.5)		39 (22.0)		2.47 (1.26-4.85)			.01			2.2 (1.05-4.62)			.04		
	10,000-19,999	120 (22.3)		68 (18.8)		52 (29.4)		3.25 (1.69-6.25)			<.001			2.55 (1.23-5.3)			.01		
	≥20,000	48 (8.9)		24 (6.6)		24 (13.6)		4.25 (1.94-9.32)			<.001			2.6 (1.09-6.23)			.03		
**Time from exposure to PEP initiation**																			
	Within 24 hours	370 (68.6)		245 (67.7)		125 (70.6)		1			—			—			—		
	>24 hours	169 (31.4)		117 (32.3)		52 (29.4)		0.87 (0.59-1.29)			.49			—			—		
**Drug use in the past 6 months**																			
	Rush poppers	194 (36.0)		122 (33.7)		72 (40.7)		1			.20			—			—		
	Mixed use of drugs	12 (2.2)		7 (1.9)		5 (2.8)		1.21 (0.37-3.96)			.75			—			—		
	No	333 (61.8)		233 (64.4)		100 (56.5)		0.73 (0.50-1.06)			.10			—			—		
**HIV high-risk sexual behavior during PEP treatment**																			
	No	490 (90.9)		339 (93.6)		151 (85.3)		1			—			1			—		
	Yes	49 (9.1)		23 (6.4)		26 (14.7)		2.54 (1.40-4.59)			<.001			1.96 (1.05-3.69)			.04		
**PEP completion**																			
	Yes	523 (97.0)		349 (96.4)		174 (98.3)		1			—			—			—		
	No	16 (3.0)		13 (3.6)		3 (1.7)		0.46 (0.13-1.65)			.23			—			—		
**Prior use of PEP**																			
	No	399 (74.0)		289 (79.8)		110 (62.1)		1			—			—			—		
	Yes	140 (26.0)		73 (20.2)		67 (37.9)		2.41 (1.62-3.59)			<.001			—			—		
**HIV testing after completing PEP**																			
	Yes	455 (84.4)		303 (83.7)		152 (85.9)		1			—			—			—		
	No	84 (15.6)		59 (16.3)		25 (14.1)		0.85 (0.51-1.40)			.51			—			—		

^a^PrEP: preexposure prophylaxis.

^b^PEP: postexposure prophylaxis.

^c^Adjusted for region, age, education, average monthly income, HIV high-risk sexual behavior during PEP treatment, and prior use of PEP.

^d^Data not available.

^e^1 RMB=US $0.14.

## Discussion

### Principal Findings

This study is the first to provide a web-based PEP provision model, and assess the uptake and outcomes of online PEP use in China. It demonstrated the feasibility of online PEP service provision as an HIV prevention option among key populations, especially Chinese MSM, with no seroconversions observed during the study period. Unlike other studies that focused mostly on PEP awareness in China, this program described the PEP service model of HeHealth, explored characteristics of online PEP recipients, and evaluated the process of PEP use in the real world, including HBV/HCV/syphilis testing prior to PEP, the average time it took to access PEP from exposure, service package delivery, and PEP drug combinations. We found high completion rate and adherence to the 28-day PEP regimen, and the PEP service delivered in this program was shown to be safe and effective.

Although there are accumulating data on PEP need among MSM [24,41], characteristics common to PEP recipients in China have not been well described yet. In our study, most individuals seeking online PEP service were MSM, which may relate to the popularity of HeHealth among Blued users. The study revealed that most of them were gay, relatively younger, and with higher education and higher monthly income. These findings contribute to the small body of literature on PEP use among Chinese MSM including identifying demographic characteristics of these primary users.

In this study, among those who received PEP, as high as 60.7% (327/539) were classified as having had only relatively low-risk exposures, suggesting that PEP was sometimes overprescribed to those with a low risk of HIV infection. This may reflect different perceptions in HIV infection risk between users and health providers. Specifically, individuals were often anxious and nervous after risky behaviors that possibly overwhelmed rational thinking. They tend to demand medicines strongly and were unwilling to take any more risks [42,43]. In view of this, it is understandable that doctors usually have to prescribe PEP medicines if exposers strongly demand them despite their low transmission risk. Meanwhile, it is suggested that doctors may need to reconsider the specific scenario of those self-reported “low-risk” behaviors to help confirm individuals’ actual risks because some generally low-risk exposures, such as oral sex, may have a high risk of HIV infection under certain conditions [44]. In addition, more elaborate guidelines on PEP service provision should be further developed, which could help optimize communication on risk-appropriate PEP treatment between health providers and patients [45].

Our program showed that the vast majority of PEP users (537/539, 99.6%) could initiate PEP request within 72 hours via an internet medical platform and over one-half (370/539, 68.6%) were within 24 hours of exposure. The time from deciding to taking PEP to submitting medication requests online and using the medicines were 1 and 6 hours, respectively, highlighting the convenience of seeking online PEP services and the importance of flexible time when using online PEP after high-risk exposure.

Without an internet medical platform, individuals have to first recognize their potential risk of exposures, make decisions on seeking PEP service with great uncertainty, find the designated hospitals, visit the hospital during its working hours, get prescription from doctors, and obtain medicines; importantly, all of these activities should be completed within 72 hours of exposure, reflecting the inherent limitations of PEP service [19,46]. Moreover, as the effectiveness of PEP largely depends on timeliness of regimen initiation, to better improve the online PEP service model, future educational campaigns should focus more on ways to shorten the time from exposure to decision making on PEP use, to help patients initiate PEP treatment as early as possible because this not only helps reduce anxiety and fear among patients, but also is beneficial to ensure effectiveness of the therapeutic prevention.

In terms of serologic testing prior to PEP treatment, our study found that the positive rates of HBV, HCV, and syphilis were 2.1% (6/282), 0% (0/121), and 5.5% (15/273), respectively. The Chinese Guideline on HIV PEP required patients to test for hepatitis B infection before initiating PEP [21], and the relatively high positive rate of HBV in this study confirmed the necessity of HBV testing. It is especially important to test HBV because it related to the choice of drug regimen for HIV (PEP). Besides, it is recommended that after initiating PEP, patients infected with HBV should be closely monitored for liver function and HBV-related indicators, and referred to the clinic of liver diseases for planned discontinuation of medication(s) [21]. In this study, none tested positive for hepatitis C, and the Chinese sentinel surveillance data also showed a low prevalence of HCV among MSM, indicating that HCV testing before initiating PEP may not be a necessity if HCV testing is unavailable. In terms of syphilis testing, considering that PEP treatment does not protect against sexually transmitted infections and the high positive rate among individuals who sought PEP services, our study suggests that syphilis screening should continue to be strengthened before initiating PEP.

In this study, 97.0% (523/539) of PEP users presumably completed the full 28-day course of medication, and this rate was higher than the completion rates in 2 other Chinese studies, where 86% [47] and 92.2% [41] of PEP users completed the 28-day course of medication, respectively. Furthermore, a systematic review and meta-analysis of 97 studies worldwide showed an even lower rate of PEP completion (67.2%; 95% CI 59.5%-74.9%) [48]. The high adherence rate of our study might be related to systematic follow-up services provided by the platform, demonstrating the value of flexibility and the potential to improve adherence via HeHealth. Further studies are, however, needed to understand specific correlates of high adherence rate observed in our study.

Over one-quarter of PEP users in this study reported prior PrEP usage, which was higher than that indicated by current limited data (12.1%) [41]. The reasons for this might include higher health awareness or more convenient access to HIV preventative services via the internet medical platform. Meanwhile, 9.1% (49/539) of the individuals continued to engage in high-risk sexual behaviors even while using PEP, which was also seen in other studies [42,49,50], and all these indicated an urgency to transition to PrEP after the PEP course, which was also specified in the Chinese guideline (ie, those who used PEP 2 times within 1 year and those with persistent HIV exposure should consider PrEP). Factors associated with PrEP usage among PEP users were higher age, higher educational level, and having HIV high-risk sexual behaviors during PEP treatment. The proportion of younger participants initiating PrEP was relatively low, suggesting that as a relatively new HIV prevention strategy, PrEP has not been promoted or practiced long enough in China, and still needs continuous and repeated health education among young people. Those with higher education used PrEP more, and it may be presumably easier for people with higher education to understand PrEP, which indicates that future attention should be paid more on ensuring that people with relatively low educational level could understand PrEP better when conducting PrEP education and promotion. The higher the income, the easier it was to initiate PrEP, and thus it is suggested to reduce the price of PrEP drugs to benefit more eligible at-risk populations. Furthermore, as PEP may provide the bridge to PrEP initiation or reengagement for some individuals [42], future PrEP education and promotion must be intensified and targeted to those who are relatively younger with lower educational and income levels, to have a population-level impact on HIV prevention. As PrEP was a proactive HIV prevention strategy, continuous efforts should be made on providing HIV-risk assessment, HIV counseling and testing, and effective linkage to PrEP services among PEP users.

### Limitations

This study has limitations. First, as the study sample was recruited from an internet medical platform, it is likely that results are not generalizable to the entire MSM population, especially to those who are not aware of this form of service. Second, a high percentage of individuals that clicked on the survey invitation link did not complete the survey, indicating a likelihood of self-selection or volunteer bias. Thus, the PEP uptake situation in this study was only applicable to those internet-sensitive MSM in China. Additionally, self-reported measures such as condom use, the HIV status of the patients’ contacts, and PEP adherence are subject to recall bias. However, as the participants used their own network terminal to fill in the questionnaires anonymously, reporting bias is unlikely to be substantial. Furthermore, the drug regimens were mainly 3TC/TDF+DTG and FTC/TDF+DTG with limited drug options; however, no participants seroconverted during the study, suggesting that these regimens should be enough to prevent HIV infection. Despite the aforesaid limitations, this study was an important first step in the exploration of PEP service provision via an online medical platform in China and filled the void of information on the characteristics of online PEP users among Chinese MSM. In general, the study contributes descriptive and analytical information on the uptake and outcome of online PEP service in China.

### Conclusions

Our study advances the understanding of online PEP service provision, characteristics of online PEP recipients, and the uptake and outcomes of PEP. We demonstrated the potential to use a web-based medical platform to provide online consultation, prescription, offline drug supply chain, and online follow-up for HIV-risk populations, especially MSM in China. The 0% infection rate in this study suggests that the online PEP provision model could be a valuable risk-reduction option and an effective strategy to improve the uptake of the prevention service within China’s existing policies and practices, which should be utilized following potential exposures to HIV among MSM in China. Moreover, it is suggested that future PEP programs could take advantage of emerging internet medical platforms to expand online provision of PEP and increase the conversion from PEP to PrEP to finally promote the overall improvements in the health of Chinese MSM.

## Appendix

Multimedia Appendix 1 CHEERIES (Checklist for Reporting Results of Internet E-Surveys) checklist [50].

## Abbreviations

3TC: lamivudine
AOR: adjusted odds ratio
DTG: dolutegravir
FTC: emtricitabine
NCAIDS, China CDC: National Center for AIDS/STD Control and Prevention, Chinese Center for Disease Control and Prevention
OR: odds ratio
PEP: postexposure prophylaxis
PrEP: preexposure prophylaxis
RAL: raltegravir
TAF: tenofovir alafenamide fumarate
TDF: tenofovir disoproxil fumarate
